# Diagnostic value of CA‐153 and CYFRA 21‐1 in predicting intraocular metastasis in patients with metastatic lung cancer

**DOI:** 10.1002/cam4.2354

**Published:** 2019-06-20

**Authors:** Qi Lin, Xuan‐Yin Chen, Wen‐Feng Liu, Pei‐Wen Zhu, Wen‐Qing Shi, Biao Li, Qing Yuan, You‐Lan Min, Jia‐Ming Liu, Yi Shao

**Affiliations:** ^1^ Department of Ophthalmology The First Affiliated Hospital of Nanchang University, Jiangxi Province Ocular Disease Clinical Research Center Nanchang Jiangxi People’s Republic of China; ^2^ Department of Orthopaedic Surgery The First Affiliated Hospital of Nanchang University Nanchang Jiangxi People’s Republic of China

**Keywords:** diagnostic value, intraocular metastases, metastatic lung cancer, risk factors

## Abstract

Lung cancer is prone to metastasis to various organs. Although intraocular metastasis (IOM) occurs at a later stage than metastasis to other organs, it often adversely affects the quality of life and suggests a poor prognosis. In this study, we selected 1608 patients with lung cancer who had metastasis to at least one site and explored clinical differences between those with IOM and non‐IOM (NIOM). An independent *t* test and chi‐squared test were used to analyze the clinical features of the patients. The statistically significant parameters were analyzed by binary logistic regression to determine the risk factors for IOM. A receiver operating characteristic curve was constructed to assess their diagnostic value in IOM. The results showed that no significant differences were noted in age, gender, and pathological type between the IOM and NIOM groups. However, the IOM group had higher levels of alpha‐fetoprotein, carcinoembryonic antigen, cancer antigen (CA)‐125, CA‐153, cytokeratin fragment 19 (CYFRA 21‐1), and total prostate‐specific antigen, compared with the NIOM group. Binary logistic regression indicated that CA‐153 and CYFRA 21‐1 were risk factors for IOM in patients with MLC (*P* < 0.05). Area under the curve of CA‐153, CYFRA 21‐1 and their combination were 0.791, 0.860, and 0.872 respectively. The cutoff values for CA‐153 and CYFRA 21‐1 were 22.2 U/mL and 6.785 ng/mL. In conclusion, both CA‐153 and CYFRA 21‐1 were independent risk factors for IOM in patients with metastatic lung cancer (MLC), whereas the combination of CA‐153 and CYFRA 21‐1 assessment yields the most value in the detection of IOM in patients with MLC.

## Graphical abstract

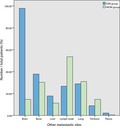

## 1 INTRODUCTION

Lung cancer is a malignant tumor that has exhibited one of the most rapid increases in morbidity and mortality. It presents a considerable threat to the well‐being of the general population. Over the past 50 years, many countries have reported a significant increase in the incidence and mortality of lung cancer.^1^ These values represent the highest incidence and mortality among all malignant tumors in men, and the second highest in women.^2^ The occurrence of lung cancer is reportedly related to smoking, occupational and environmental factors, genetic susceptibility, age, sex, and race, among other factors.^3^, ^4^, ^5^

Lung cancer can be divided into non‐small cell lung cancer (NSCLC) and small cell lung cancer. The two types differ in biology and sensitivity to chemotherapy and radiotherapy.^6^, ^7^ Generally, lung cancer is very prone to metastasis. The common metastatic sites detected clinically include the brain, lymph nodes, lung, bone, and liver. Previous studies have shown that organ metastasis indicates a poor prognosis.^8^ The median survival time of patients with NSCLC without metastasis is 21 months, whereas the median survival time of patients with metastases is 6‐7 months and 5 months in the brain and bone, respectively.^9^, ^10^ Furthermore, the overall survival for patients with lung cancer without brain metastases has been found to correlate with the number of metastatic sites.^9^

Intraocular metastasis (IOM), as a relatively rare distant metastasis of lung cancer, often occurs at a later stage than metastases to other organs and tissues. Because of its low incidence and minor early clinical symptoms, many people pay little attention to IOM. However, as it develops, the condition leads to swelling of the eyeball, pain, and vision deficiency, thereby adversely affecting the quality of life of lung cancer patients.^11^ Thus, early diagnosis of IOM is very important, which is beneficial to the early control of the disease and the improvement of the quality of life of the patients.

A tumor marker is a substance that is either synthesized and secreted through the genetic expression of cancer cells, or abnormally produced through the body's response to the occurrence and proliferation of malignant tumors. Tumor markers reflect the existence and growth of tumors. According to the essence, tumor markers can be divided into protein, carbohydrate, lipid, enzymes, hormones, polyamines, and gene products.^12^ Tumor markers mainly exist in the serum and serous effusions and can be detected by immunological, biological and chemical methods. Thus, they are easy to collect, inexpensive, and less invasive compared with computed tomography (CT) and magnetic resonance imaging (MRI) scans. In recent years, the use of tumor markers in the field of diagnosis of metastasis has made some progress.

However, it is not clear whether there are differences in biomarkers between MLC patients with or without IOM. Therefore, in this study, we focused on the diagnostic values of tumor markers when used to clarify the risk factors for IOM in patients with metastatic lung cancer.

## 2 MATERIALS AND METHODS

### 2.1 Study design

All subjects gave their informed consent for inclusion and agreed to be included in the study before participating in the study. This study was approved by the Medical Research Ethics Committee of the First Affiliated Hospital of Nanchang University. All procedures of this study were conducted in accordance with the Declaration of Helsinki. A series of consecutive patients who participated in this study were diagnosed with lung cancer between January 2002 and December 2016 and had at least one site of metastasis. The diagnosis of lung cancer was based on pathological sections obtained by surgical excision or biopsy. The diagnosis of metastasis was based on CT or MRI and biopsy. The IOM was diagnosed by CT and MRI. Patients with primary intraocular malignancies, benign intraocular tumors, and secondary lung cancer were excluded. All participants in the study were provided with the entire research design and gave informed consent.

### 2.2 Data collection

The clinical data were collected from the medical records of patients with MLC, including age, sex, histopathological type, and treatment. Some tumor markers were also detected, including calcium, hemoglobin (HB), alkaline phosphatase (ALP), alpha‐fetoprotein (AFP), carcinoembryonic antigen (CEA), neuron‐specific enolase (NSE), cancer antigen (CA)‐125, CA‐153, CA‐199, cytokeratin fragment 19 (CYFRA 21‐1), and total prostate‐specific antigen (TPSA). All clinical data were collected when the patients were diagnosed with lung cancer and metastasis.

### 2.3 Statistical analysis

We performed an independent *t* test and chi‐squared test to compare the features of age, sex, and histopathological subtypes. The differences between tumor markers in the IOM group and the non‐IOM (NIOM) group were also analyzed by an independent *t* test. Binary logistic regression models were then applied to identify the independent risk factors for IOM. A receiver operating characteristic (ROC) curve was constructed and the area under the curve (AUC) was calculated. Afterward, we used Excel 2010 software to calculate the cutoff value, sensitivity, and specificity of risk factors. A value of *P* < 0.05 indicated statistical significance. All statistical analyses were performed using the SPSS 20.0 software (SPSS, IBM, USA) and Excel 2010 software (Excel, Microsoft, USA).

## 3 RESULTS

### 3.1 Demographics and clinical characteristics

**Figure 1:**
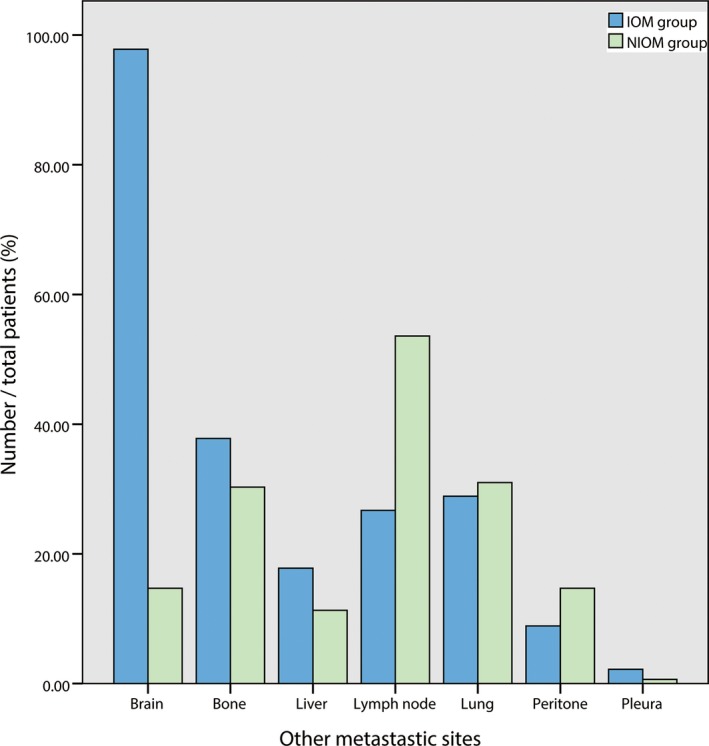
Other metastatic sites of IOM and NIOM groups. IOM, intraocular metastasis; NIOM, nonintrocular metastasis

A total of 1608 patients (1168 male and 440 female) were recruited for the study, which comprised 45 patients with IOM and 1563 patients considered NIOM cases. The average ages of the IOM and NIOM groups were 58.5 ± 1.6 and 60.0 ± 0.3 years, respectively. No significant differences (*P* > 0.05) were noted in gender and age between the IOM and NIOM groups. Regarding the histopathological subtype, adenocarcinoma was the most common type in both the IOM group and NIOM group. However, the distribution of histological types between the IOM group and NIOM group was significantly different (*P* < 0.001). More details are shown in Table 1. In addition, other metastatic sites of IOM group were brain (97.8%), bone (37.8%), liver (17.8%), lymph node (26.7%), lung (28.9%), peritoneum (8.9%) and pleura(2.2%), while metastatic sites of NIOM group were brain (14.7%), bone (30.3%), liver (11.3%), lymph node (67.6%), lung (31.0%), peritoneum (14.7%), and pleura(0.64%). More details are shown in Table 2 and Figure 1.

**Table 1:** The clinical characteristics of patients with MLC

Patient characteristics	IOM group (n = 45)	NIOM group (n = 1563)	*P* value
Gender^a^			
Male	34	1134	0.478
Female	11	429	
Mean age^b^	58.5 ± 1.6	60.0 ± 0.3	0.374
Histopathological type^a^			
Squamous cell carcinoma	7	507	<0.001
Adenocarcinoma	33	727	
Large cell carcinoma	0	29	
Small cell lung cancer (SCLC)	3	194	
Other NSCLC	1	17	
Unknown	1	89	
Treatment			
Surgery	6	272	
Chemotherapy	40	1018	
Radiotherapy	11	168	
Symptomatic treatment	3	332	

Abbreviations: IOM, intraocular metastasis; MLC, metastatic lung cancer; NIOM, non‐intraocular metastasis; NSCLC, non‐small cell lung cancer; SCLC, small cell lung cancer.

a Chi‐square test was applied.

b Student‐*t* test was applied. *P* < 0.05 was thought to be statistical significant.

**Table 2:** Other metastatic sites of IOM and NIOM groups

Sites	IOM	NIOM
Brain	44 (97.8%)	230 (14.7%)
Bone	17 (37.8%)	474 (30.3%)
Liver	8 (17.8%)	176 (11.3%)
Lymph node	12 (26.7%)	1057 (67.6%)
Lung	13 (28.9%)	485 (31.0%)
Peritoneum	4 (8.9%)	230 (14.7%)
Pleura	1 (2.2%)	10 (0.64%)

Abbreviations: IOM, intraocular metastasis; NIOM, nonintraocular metastasis.

### 3.2 Differences in the clinical features and risk factors of ocular metastasis

No significant differences were noted in the levels of calcium, HB, ALP, NSE, and CA‐199 between the IOM and NIOM groups (*P* > 0.05). However, increased levels of AFP, CEA, CA‐125, CA‐153, CYFRA 21‐1, and TPSA were observed in the IOM group compared with the NIOM group (*P* < 0.05). Detailed results are presented in Table 3. The results of the binary logistic regression model showed that CA‐153 and CYFRA 21‐1 could be considered independent risk factors of IOM in patients with MLC. More details are shown in Table 4.

**Table 3:** Differences of tumor markers between MLC patients with and without IOM

Tumor markers	IOM group	NIOM group	*t*	*P* value
Calcium (mmol/L)	2.30 ± 0.03	2.23 ± 0.01	1.907	0.057
HB (g/L)	114.11 ± 3.03	117.50 ± 0.48	−1.172	0.241
ALP (U/L)	110.33 ± 8.41	95.45 ± 2.50	1.695	0.096
AFP (ng/mL)	3.13 ± 0.32	2.33 ± 0.04	2.497	0.016
CEA (ng/mL)	277.42 ± 84.39	60.12 ± 7.63	2.564	0.014
NSE (μg/L)	27.67 ± 2.36	30.03 ± 1.20	−0.333	0.740
CA‐125 (U/mL)	350.09 ± 71.42	84.32 ± 5.18	3.711	0.001
CA‐153 (U/mL)	87.96 ± 17.70	22.63 ± 0.92	3.686	0.001
CA‐199 (U/mL)	146.37 ± 48.17	67.86 ± 14.71	0.902	0.367
CYFRA 21‐1(ng/mL)	35.91 ± 3.52	12.07 ± 0.92	4.397	＜0.001
TPSA (ng/L)	4.13 ± 0.36	1.79 ± 0.11	3.515	＜0.001

Note

Independent samples‐*t* test was applied. *P* < 0.05 represented statistical significant.

Abbreviations: AFP, alpha‐fetoprotein; ALP, alkaline phosphatase; CA, cancer antigen; CEA, carcinoembryonic antigen; HB, hemoglobin; IOM, intraocular metastasis; MLC, metastatic lung cancer; NIOM, nonintraocular metastasis; NSE, neuron‐specific enolase; TPSA, total prostate‐specific antigen.

**Table 4:** Risk factors of IOM in patients with MLC

Factors	B	Exp(B)	OR (95% CI)	*P*
CEA	0.000	0.999	0.999‐1.000	0.060
CA‐125	0.000	0.999	0.098‐1.000	0.093
CA‐153	−0.009	0.991	0.987‐0.995	<0.001
CYFRA 21‐1	−0.007	0.993	0.989‐0.997	0.001
TPSA	−0.029	0.971	0.941‐1.003	0.074

Note

Binary logistic Analysis was applied. *P* < 0.05 represented statistical significant.

Abbreviations: B, coefficient of regression; CA, cancer antigen; CEA, carcinoembryonic antigen; CI, confidence interval; IOM, intraocular metastasis; MLC, metastatic lung cancer; OR, odds ratio; TPSA, total prostate‐specific antigen.

### 3.3 The cutoff value, AUC, sensitivity, and specificity of CA‐153 and CYFRA 21‐1 for the diagnosis of IOM

**Figure 2:**
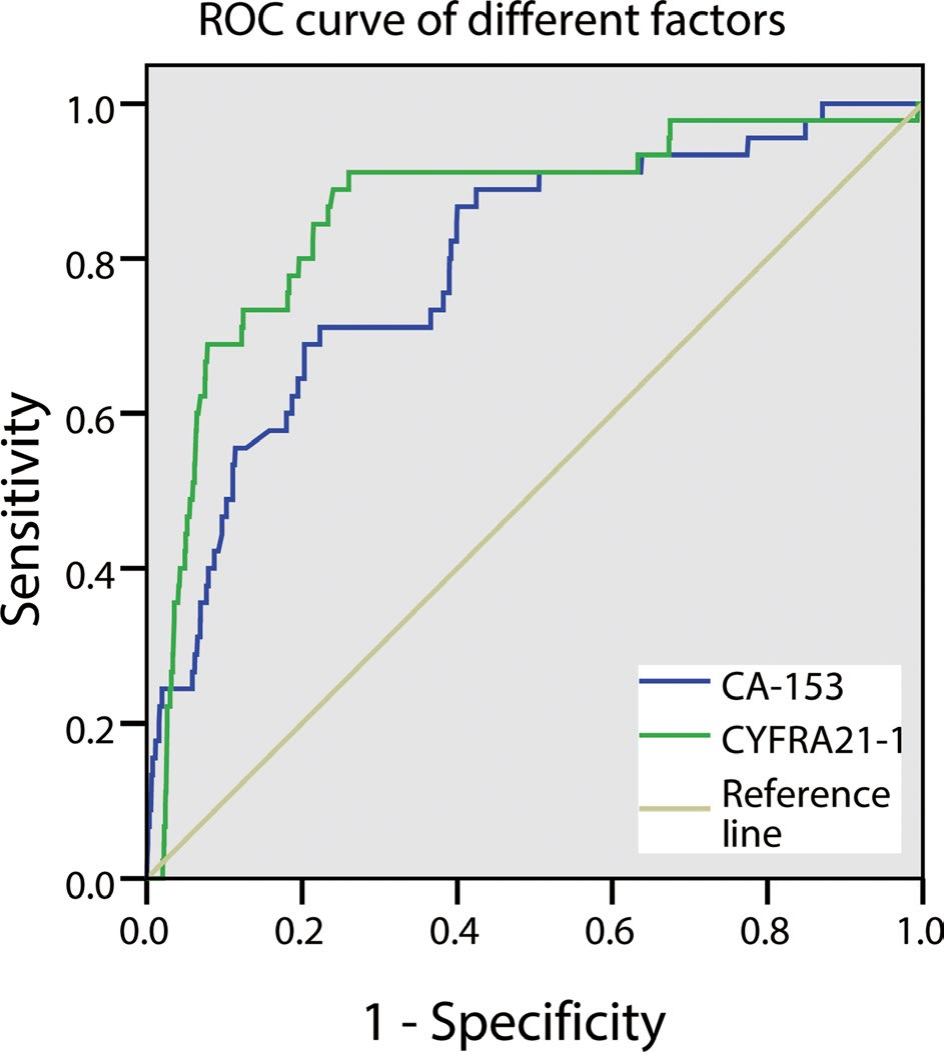
The ROC curves of risk factor for detecting IOM in MLC. ROC curves of CA‐153 and CYFRA21‐1 as single risk factor of IOM. CA, cancer antigen; IOM, intraocular metastasis; MLC, metastatic lung cancer; ROC, receiver operating characteristic

**Figure 3:**
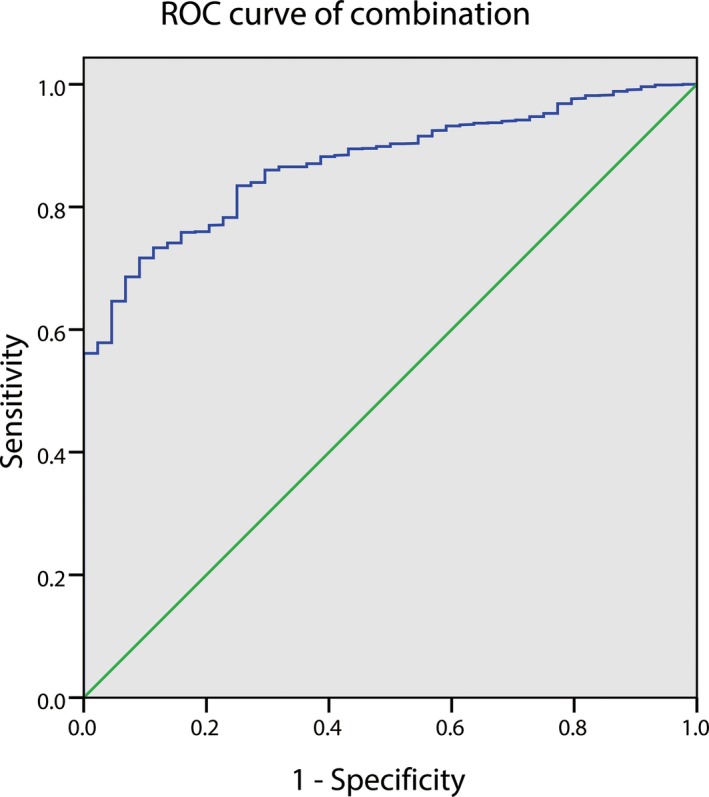
The ROC curves of combination of CA‐153 and CYFRA21‐1 for detecting IOM in MLC. CA, cancer antigen; IOM, intraocular metastasis; MLC, metastatic lung cancer; ROC, receiver operating characteristic

The ROC curve showed that the AUC for CA‐153 and CYFRA 21‐1 were 0.791 and 0.860, respectively. The AUC for CYFRA 21‐1 was the highest (Figure 2). The cutoff value of CA‐153 and CYFRA 21‐1 were 22.2 U/mL and 6.785 ng/mL, respectively (Table 5). And the corresponding sensitivity and specificity were also shown in Table 4. Furthermore, we made combination of the two factors, and Figure 3 shows the ROC curves for CA‐153 + CYFRA 21‐1. The results showed a higher AUC value for the combination of CA‐153 + CYFRA 21‐1 than that for each individual factor.

**Table 5:** The cutoff value, sensitivity, specificity, and AUC for a single risk factor in predicting IOM in MLC patients

Factor	Cutoff value	Sensitivity (%)	Specificity (%)	AUC	*P*
CA‐153 (U/mL)	22.2	71.1	77.7	0.791	<0.001
CYFRA 21‐1(ng/mL)	6.785	91.1	74	0.860	<0.001
CA‐153 + CYFRA 21‐1	—	71.7	90.9	0.872	<0.001

Note

Sensitivity and specificity were obtained at the point of cutoff value. *P* < 0.05 represented statistical significant.

Abbreviations: AUC, area under the curve; CA, cancer antigen; CI, confidence interval; IOM, intraocular metastasis; MLC, metastatic lung cancer.

## 4 DISCUSSION

Lung cancer has the highest incidence of malignant tumors in China, and the highest mortality rate among all types of malignant tumors.^13^ Distant metastasis is one of the main characteristics of malignant tumors. Many patients with lung cancer in China have already developed local or distant metastases at the time of diagnosis. Although IOM is rare, it is indicative of a poor prognosis. Thus, an early diagnosis of IOM is crucial. Consistent with Xu's study,^14^ most patients with IOM concurrently had metastases at other sites, including the brain, bone, and liver. Therefore, in this study, we recruited patients with lung cancer, who had metastases to at least one site, to evaluate the diagnostic value of tumor markers in predicting IOM in patients with MLC, and to avoid bias in the results.

At present, tumor markers are mainly used in the diagnosis, and evaluation of curative effects, recurrence, and prognosis of malignant tumors.^15^ Park et al^16^ retrospectively analyzed the medical records of 156 patients (79 hepatocellular carcinoma 77 liver cirrhosis) and found that AFP was high in patients with hepatocellular carcinoma. In addition, the AUC of AFP was 0.765, so they came to the conclusion that AFP was a useful single biomarker for diagnosing hepatocellular carcinoma. In another study,^17^ CA**‐**125 and CEA were found to be associated with the clinical stage of lung cancer and CYFRA 21‐1and NSE can be used to monitor the effect of chemotherapy. Besides, serum levels of CA**‐**199 had great value for predicting prognosis in pancreatic cancer patients.^18^ What is more, tumor markers can be used to predict cancer metastasis, which is of great significance. For, example, Huang et al^19^ observed that ALP and HB were risk factors for bone metastases in patients with bladder cancer. And CA**‐**125 might play an important role in tumor metastasis, and could be thought as an independent risk factor for bone metastases in patients with lung cancer.^20^

Previous studies about the risk factors of clinical parameters for distant metastases of lung cancer are listed in Table 6, which also indicates the possibility of tumor marker levels to predict tumor metastases and make up the limitations for CT, PET/CT and MRI.

**Table 6:** The risk factors of metastases of lung cancer

Author	Year	Histopathological type	Metastatic sites	Risk factor
Pollán et al^21^	2003	NSCLC	NS	CA‐125
Oshiro et al^22^	2004	Adenocarcinoma	Liver	AFP
Cabreraalarcon et al^23^	2011	NS	NS	CYFRA 21‐1
Lee et al^24^	2012	NSCLC	Brain	CEA
Chen et al^25^	2015	NS	Lymph node	CYFRA 21‐1, CEA
Chen et al^26^	2015	NSCLC	Brain	NSE
Zhou et^20^	2017	NS	Bone	CA‐125, ALP
Morita et al^27^	2019	NSCLC	Intertrabecular vertebral	CEA

Abbreviations: AFP, alpha‐fetoprotein; CA, cancer antigen; CEA, carcinoembryonic antigen; NS, not specific; NSCLC, non‐small cell lung cancer; NSE, neuron‐specific enolase.

After analyzing the clinical data of 1608 patients with MLC, we found that the concentration of AFP, CEA, CA‐125, CA‐153, CYFRA 21‐1, and TPSA were significantly elevated in patients with IOM. However, in lung cancer, AFP is associated with liver metastasis [22]. Thus, we excluded AFP from the tumor markers under consideration. According to the results of the binary logistic regression model, CA‐153 and CYFRA 21‐1 might be independent risk factors for IOM in patients with MLC (*P* < 0.001).

CA153 was first found on the membrane of breast cancer cells with a relative molecular weight of 4000.^28^, ^29^ Its structure consists of a membrane region, an intracellular region and an extracellular region rich in glycosyl groups. CA153 can be detached from the cancer cell membrane and released into the blood. And the antigen determinant of its extracellular domain can be determined by specific binding of monoclonal antibodies. Abnormal levels of CA153 can also exist in lung cancer, endometrial cancer, and gastrointestinal cancer.^30^, ^31^, ^32^ In addition, it is useful in the prediction of bone metastasis and distant metastases in patients with breast cancer.^33^, ^34^ CYFRA 21‐1 is a new tumor marker developed in recent years. It is a fragment of cytokeratin 19 which is produced during the differentiation of cancer cells. Cytokeratin 19 is a characteristic protein component of epithelial cell filament, which exists in many normal epithelial tissues.^35^ When the epithelial cells transformed into tumors, the cytokeratin structure remained unchanged but increased in content. Due to the necrosis and dissolution of the tumor cells, the soluble fragment CYFRA 21‐1 could be released into the blood. According to previous studies, CYFRA 21‐1 is associated with lung cancer, tumors of the urinary system, and gastrointestinal tract, as well as gynecological tumors.^36^, ^37^, ^38^, ^39^ Molina et al^40^ reported that serum CYFRA 21‐1 levels differ between different stages of lung cancer, indicating that high levels of CYFRA 21‐1 are associated with an advanced stage of the tumors. Moreover, Choi et al^41^ found that CYFRA 21‐1 is a tumor biomarker for axillary lymph node metastasis in breast cancer. Furthermore, in lung cancer, CYFRA 21‐1 is associated with distant metastasis.^23^ Based on the respective analyses of CA‐153 and CYFRA 21‐1 levels, and the high AUC of the two biomarkers, we concluded that they are both independent risk factors for IOM in MLC.

The results based on the cutoff values showed that CA‐153 > 22.2 U/mL and CYFRA 21‐1 > 6.785 ng/mL were features of IOM in patients with MLC. A higher AUC was observed for CYFRA 21‐1, suggesting its higher diagnostic value in predicting IOM. In this study, we also analyzed the accuracy of diagnosis based on a combination of these risk factors. The results showed that the combined CA‐153 + CYFRA 21‐1 showed higher accuracy in predicting IOM. In addition, the combined CA‐153 + CYFRA 21‐1 showed both high sensitivity and specificity.

Although the results were significant, our study still had some limitations. First, the sample size was relatively small in the IOM group, which meant the outcome was insufficiently convincing. Second, all participants were from the same region and hospital. Third, owing to the long duration under consideration, some data were unknown, and this may have affected the results. Thus, it is necessary to verify the results of this study with future prospective data and multiple‐center analysis.

In conclusion, based on this study of 1608 patients with MLC, the results suggest that the serum concentration of CA‐153 and CYFRA 21‐1 were independent risk factors for IOM. In addition, the combination of CA‐153 and CYFRA 21‐1 likely has higher accuracy in predicting IOM.

## ETHICS APPROVAL AND CONSENT TO PARTICIPATE

Not applicable.

## CONSENT FOR PUBLICATION

Not applicable.

## CONFLICT OF INTEREST

This was not an industry supported study. The authors report no conflict of interest in this work.

## AUTHORS' CONTRIBUTIONS

Qi Lin, Xuan‐Yin Chen and Wen‐Feng Liu were responsible for conceiving and designing the work, acquiring data and writing the manuscript; Pei‐Wen Zhu played an important role in interpreting the results; Wen‐Qing Shi helped the first two authors in writing the manuscript; Biao Li and Qing Yuan helped acquire data and gave some advice; You‐Lan Min helped perform the analysis with constructive discussions; Yi Shao and Jia‐Ming Liu helped design the work and approved the final version.

## Data Availability

All data generated or analyzed during this study are included in this published article.
